# Evaluation of anti-oxidant and antimelanogenic effects of the essential oil and extracts of *Rosa *×* damascena* in B16F10 murine melanoma cell line 

**DOI:** 10.22038/IJBMS.2023.69734.15182

**Published:** 2023

**Authors:** Elham Hadipour, Mona Rezazadeh Kafash, Seyed Ahmad Emami, Javad Asili, Zahra Boghrati, Zahra Tayarani-Najaran

**Affiliations:** 1 Department of Biology, Faculty of Science, University of Guilan, Rasht, Iran; 2 Targeted Drug Delivery Research Center, Pharmaceutical Technology Institute, Mashhad University of Medical Sciences, Mashhad, Iran; 3 Department of Traditional Pharmacy, School of Pharmacy, Mashhad University of Medical Sciences, Mashhad, Iran; 4 Department of Pharmacognosy, School of Pharmacy, Mashhad University of Medical Sciences, Mashhad, Iran

**Keywords:** B16F10 cell line, Melanogenesis, * Rosa* × *damascena*, Reactive oxygen species, Tyrosinase

## Abstract

**Objective(s):**

*Rosa *× *damascena* Herrm. belonging to the Rosaceae family has demonstrated anti-inflammatory and anti-oxidant effects previously. Excessive production of free radicals and activation of tyrosinase enzyme caused by UV induces excessive concentration of melanin pigment and skin spots in the long term. Therefore, finding natural sources with anti-oxidant and antityrosinase effects helps to regulate the melanogenesis process. In the current research, we investigated the antimelanogenic, anti-oxidant, and anti-tyrosinase effects of its essential oil, methanol extract (MeOH), and different fractions including n-hexane, dichloromethane (CH_2_Cl_2_), n-butanol (BuOH), ethyl acetate (EtOAc), and H_2_O of *R*. × *damascena* in B16F10 cell line.

**Materials and Methods:**

For this purpose, impacts of extracts and essential oil of *R.* × *damascena* were investigated on cell viability, cellular tyrosinase, melanin content, mushroom tyrosinase, reactive oxygen species (ROS) production, as well as the amount of tyrosinase protein in the B16F10 murine melanoma cell line.

**Results:**

Essential oil, MeOH, and different fractions of *R. *× *damascena* were not cytotoxic on B16F10 cells. However, they had significant reducing effects on mushroom tyrosinase activity, melanin content, and ROS production. Also, there is a significant decrease in tyrosinase protein levels at 200 µg/ml but not at other concentrations.

**Conclusion:**

Therefore, the essential oil, MeOH, and different fractions of *R. *× *damascena* had promising antimelanogenic activity via repression of mushroom tyrosinase activity and ROS production.

## Introduction

*R. × damascena* which is also called Damask rose (1) holds some varieties which are very important for essential oil production (2). Several studies show that this plant has anti-human immunodeficiency viruses (HIV), anti-viral effects (3), antibacterial (4), anti-inflammatory, and anti-oxidant (5, 6), as well as hypnotic (7) and bronchodilatory properties (8). *R. × damascena* contains compounds such as carboxylic acids, linalool C10H180O, eugenol, citronellol C10H20, farnesol, nerol C10H18, terpenes, myrcene, quercetin, kaempferol, and vitamin C. The oil extract of this plant contains 16–35% citronellol, 8–30% geraniol, 4–10% nerol, 4–16% nonadecane, 3–8% heneicosane, and 1–3% linalool. Also, compounds such as isoquercitrin, afzelin, quercetin gentabiosid, and cyanidin-3-O-beta-glucoside have been identified in the rosebud (9. Its hydro-alcoholic extract of petals and essential oil have significant scavenging effects on oxidative agents compared with the standards (6). In general, the medicinal functions of the rosacea family which include anti-oxidant, free radical absorber, anticancer, anti-inflammatory, and anti-depressant activity are largely dependent on phenolic compounds, (10). Melanin pigmentation in the skin has protective effects against the rays of the sun (11) besides, it is responsible for thermoregulation in lower vertebrates, camouflage, and mimicry (12). The production of excess melanin pigments leads to some skin problems, including melasma, and sunspots (13). Melanin production occurs in a part of the melanocyte called melanosome by the amino acid tyrosine. Tyrosinase is involved not only in the melanogenesis process in humans and animals but also in food browning. Therefore, inhibition of tyrosine production has important medical use in the treatment of the production of excess melanin pigments and is widely used in the cosmetics industry in skin whitening and depigmentation products, as well as in food processing and agriculture (13). The leading cause of skin hyperpigmentation is oxidative stress mostly caused by ROS such as ONOO-, ·O_2_, and NO· (14, 15). As a result, compounds with anti-oxidative properties might be useful as melanin synthesis regulators and antimelanogenesis agents (16). The *Rose* belongs to the *Rosacea* family and has over 200 species around the world (17). Some literature has been published on the biological functions of* R.* *× damascena*, however, there are few studies on the inhibitory effects of *R.* *× damascena* on melanin production. It was shown that the ethanol extract of the *Rosa gallica* petal has anti-aging and anti-oxidant properties due to its high flavonoid content (18). Additionally, it has been demonstrated that the extracts and compounds isolated from the Rosa chinensis cv. ‘JinBian’ have anti-aging, anti-tyrosinase, and antibacterial properties, and these natural substances can be used in the production of anti-aging, skin lightening, and antibacterial products (19). Similarly rose petal extract has whitening and anti-wrinkle effects on the skin through activation of mitogen-activated protein kinase (MAPK) and inhibition of matrix metalloproteinase 1 (MMP-1) (20). On the other hand, the anti-tyrosinase activity of the methanol extract of Rosa indica has been reported (21). Thus, here we investigated the anti-oxidant and inhibitory activity of essential oil, MeOH, and different fractions of *R.* *× damascena *against melanin production in the B16F10 murine melanoma cell line. 

## Materials and Methods

***Materials***

The aerial parts of *R.* *× damascena *were collected in May 2014 from Kashan, Isfahan province, Iran. The plant material has been identified and is kept in the Herbarium of the Faculty of Pharmacy, Mashhad Medical University. Resazurin, 2’,7’- dichlorodihydrofluorescein diacetate (DCFH-DA), Roswell park memorial institute medium 1640 (RPMI medium), L-DOPA, Kojic acid, mushroom tyrosinase, and QuantiPro™ BCA Assay Kit were purchased from Sigma (Germany); rabbit anti-tyrosinase antibody, β-Actin (13E5) and anti-rabbit IgG HRP-linked antibody were purchased from Cell Signaling Technology (USA); Fetal bovine serum (FBS) and penicillin-streptomycin (PS) were purchased from Gibco (USA); methanol, n-hexane, CH_2_Cl_2_, BuOH, and EtOAc were from Merk (Germany); B16F10 melanoma cells were purchased from Pasteur Institute (Iran).

***Preparation of plant material***

The aerial parts of *R.* × *damascena *were air-dried and powdered. Then 100 g of this powder was extracted with 1.5-liter methanol (90%) at room temperature by percolation method. The process was continued until the obtained extract was colorless. The extract was then concentrated under reduced pressure (Rotary evaporator) and dried completely using a freeze dryer. The weight of the obtained dried extract was 27.5 g. In order to fractionate the extract and abstract chlorophyll and lipophilic components, it was suspended in 90% methanol (MeOH) and successively partitioned n-hexane, CH_2_Cl_2_, BuOH, EtOAc, and H_2_O. Different phases were separated according to polarity. Eventually, the obtained extracts were evaporated by a rotary evaporator and dried completely by freeze drying. The essential oil of rose was obtained by a specified hydro-distillation method and donated by Dr A Ebrahimabadi, Department of Essential Oils Research Center, University of Kashan, Kashan, Iran (22). 

***Gas-chromatography (GC) and GC-MS***

The GC-FID analyses were performed using a Varian CP-3800 GC apparatus with a CP-Sil 8CB column (30 m x 0.25 mm i.d., 0.12 µm film thickness) and an FID detector. Oven temperature 50 °C (5 min), 50 °C–250 °C (3 °C /min), 250 °C (5 min); injector temperature 250 °C; volume injection, 1 µl; split ratio, 1:5; carrier gas N2 at 2.0 ml/min; detector temperature, 280 °C.

The GC-MS analyses were done using an Agilent 5975 apparatus with an HP-5ms column (30 m x 0.25 mm i.d., 0.25 µm film thickness) interfaced with a quadruple mass detector and a computer equipped with Wiley 7n.l library; oven temperature 50 °C (5.0 min), 50 °C–250 °C (3 °C/min), 250 °C (5.0 min); injector temperature 250 °C; volume injection, 0.1 µl; split ration, 1:50; carrier gas Helium at 1.1 ml/min; ionization potential,70 eV; ionization current, 150 µA; ion source temperature, 250 °C; mass range, 35–465 mui.

Identification of individual compounds was made by comparison of their mass spectra and retention indices (RI) on their Retention Kovats Index, calculated in relation to the retention time of a series of n-alkanes (C8-C20) and with those authentic samples and those given in the literature (23). Quantification of the relative amount of the individual components was performed according to the area percentage method in GC-FID spectra without consideration of the calibration factor.

***Cells and cell culture***

B16F10 melanoma cells were cultured in a 37 °C incubator with 5% CO_2_ and 90% humidity for 24 hr. RPMI 1640 plus 10% FBS and 1% PS was used as culture media. Kojic acid (2 and 4 mM) was used as a positive control [1). 

***Cell viability assay***

The Resazurin method was done to measure the cell viability. 10^5^ cells were treated with *R. × damascena *essential oil, MeOH, and different fractions (0.2–200 µg/ml) for 24 hr and the absorbance was compared after addition of resazurin to each well (24).

***Melanin content assay***

10^5^ cells were treated with *R. × damascena *essential oil, MeOH, and different fractions (0.2–200 µg/ml). After 24 hr, the purified melanin was extracted and its content was determined by reading the absorbance at 405 nm (11). 

***Mushroom tyrosinase activity assay***

*R. × damascena *essential oil, MeOH, and different fractions (0, 10, 100, and 1000 µg/ml) were added to each well of 96-well microplates. The next day, cells were treated with 160 µl L-DOPA and 20 µl of mushroom tyrosinase, 30 min before measurement of absorbance at 457 nm using enzyme-linked immunosorbent assay (ELISA) Reader (Awareness, USA) (11). 

***Cellular tyrosinase activity assay***

10^5^ cells were treated with *R. × damascena *essential oil, MeOH, and different fractions (0.2–200 µg/ml) for 24 hr. Afterward, cells were lysed with 50 µl of lysis buffer and mixed with 30 µl of L-DOPA. After 2 hr, the absorbance of samples was read at 457 nm with an ELISA Reader (11). 

***Cellular ROS level determination***

10^4^ cells were treated with *R. × damascena *essential oil, MeOH, and different fractions (0.2–200 µg/ml) for 24 hr. After adding 2’, 7’-dichlorofluorescein diacetate (DCFH-DA) (10 μl) to each well, fluorescent emission of samples were read at 525 nm with Synergy H4 Hybrid Multi-Mode Microplate Reader (BioTek, Winooski, USA) (11). 

***Western blotting***

Cells were treated with *R. × damascena *essential oil (0.2–200 µg/ml) for 24 hr. The next day, they were lysed in a buffer, and 50 µg of proteins was transferred to a polyvinylidene difluoride membrane after separation in gel electrophoresis with 10% SDS-polyacrylamide. After, the membrane was exposed to primary antibodies: rabbit anti-tyrosinase antibody (1:300), β-actin (13E5), and then to anti-rabbit IgG (1:2000) as a secondary antibody. Finally, bands were detected by a Gel-pro Analyzer V. 6.0 image analysis software (Media Cybernetics, InG, Bethesda, MD, USA). β-Actin was used as internal control (25). 

***Statistical analysis***

Mean±SD of all data was measured and significant differences (*P*<0.05) between groups were analyzed with Graph Pad Prism®: ANOVA and Dennett’s *post hoc* test

## Results

***Essential oil composition***

The chemical composition (99.8%) of the essential oils of *R. × damascena *aerial parts identified 63 components as volatiles using GC-MS analysis (Table 1).

***Effect of R. × damascena essential oil, MeOH, and different fractions on cell viability of B16F10 cells***

**Figure 1:**
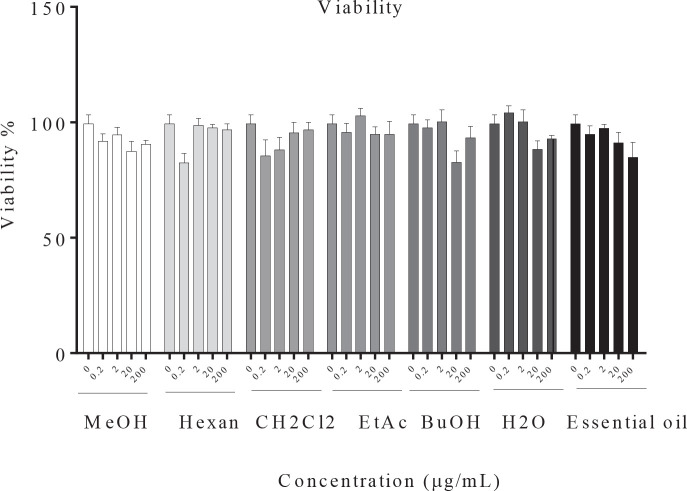
Effect of *Rosa *× *damascena* essential oil, MeOH, and different fractions on cell viability in B16F10 melanoma cells. The viability of B16F10 melanoma cells was evaluated after treatment with essential oil, MeOH, and different fractions of *R.* × *damascena* (0.2–200 µg/ml), Data are expressed as mean±SD. *P<0.05 compared with control

It was shown in the resazurin assay that essential oil, MeOH, and different fractions of *R. × damascena* have no significant cytotoxic effect (Figure 1). Hence, *R. × damascena *does not have cytotoxic effects on B16F10 melanoma cells.

***Effect of R. × damascena essential oil, MeOH, and different fractions on melanin production***

**Figure 2:**
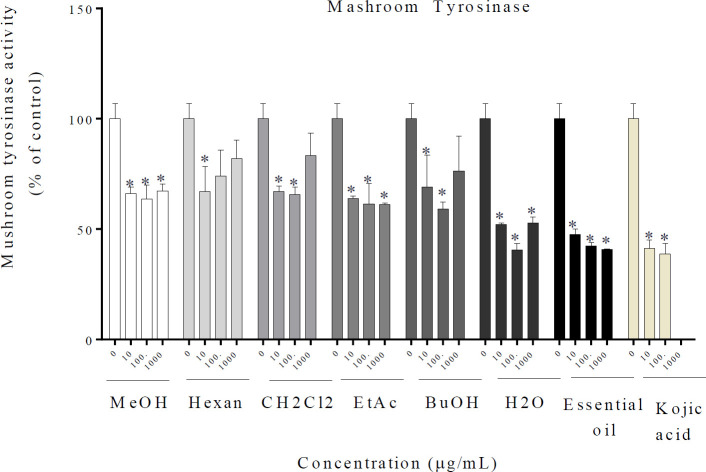
Effect of *Rosa* × *damascena* essential oil, MeOH, and different fractions on melanin content in B16F10 melanoma cells. B16F10 cells were treated with essential oil, MeOH, and different fractions of *R.* × *damascena* (0.2–200 µg/ml). Data are expressed as mean±SD. *P<0.05 compared with control

It was seen that melanin levels were reduced by *R. × damascena *essential oil at concentrations of 2, 20, and 200 µg/ml. On the other hand, MeOH and fractions had no significant reducing effect on melanin content (Figure 2).

***Effect of R. × damascena essential oil, MeOH, and different fractions on mushroom tyrosinase activity***

**Figure 3:**
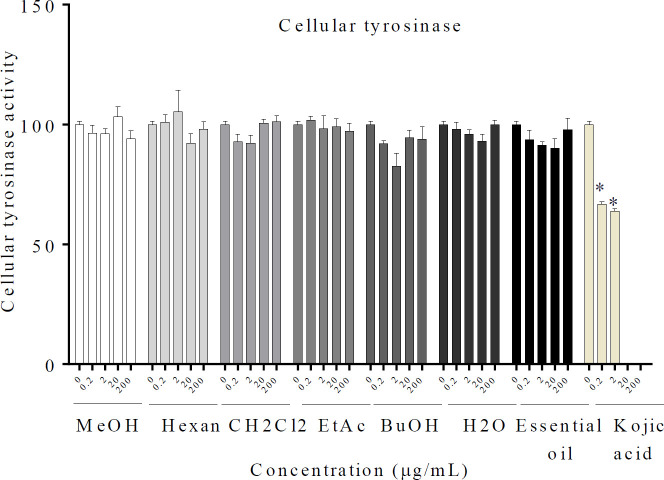
Effect of *Rosa* × *damascena* essential oil, MeOH, and different fractions on mushroom tyrosinase in B16F10 melanoma cells.* R. *× *damascena* essential oil, MeOH, and different fractions and 2, 4 mM of Kojic acid were treated with mushroom tyrosinase and L-DOPA. Data are expressed as mean±SD. *P<0.05 compared with control

The essential oil, MeOH, and different fractions significantly reduced the activity of mushroom tyrosinase. The obtained results are presented in Figure 3. 

***Effect of R. × damascena essential oil, MeOH, and different fractions on cellular tyrosinase activity of B16F10 cells***

**Figure 4:**
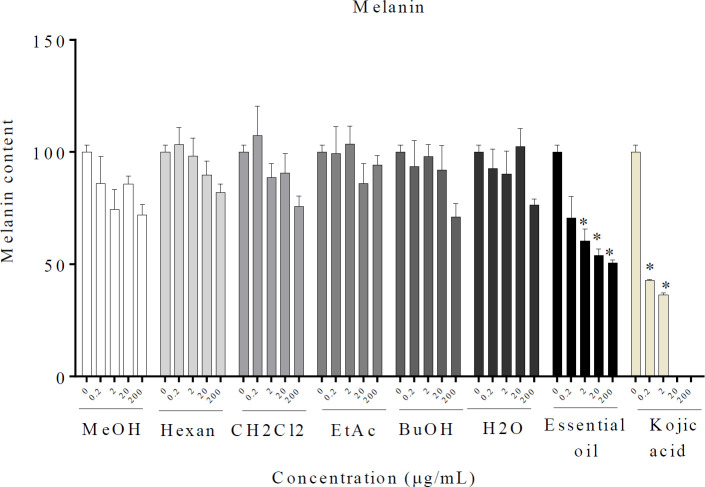
Effect of *Rosa* × *damascena* essential oil, MeOH, and different fractions on cellular tyrosinase in B16F10 melanoma cells. After treatment of B16F10 melanoma cells with essential oil, MeOH, and different fractions of *R.* × *damascena* (0.2–200 µg/ml), cellular tyrosinase activity was assessed. Data are expressed as mean±SD. *P<0.05 compared with control

Looking at Figure 4, it is apparent that the essential oil, MeOH, and different fractions displayed no inhibitory effect. So, it has not directly affected cellular tyrosinase activity. Also, Kojic acid inhibited tyrosinase activity by 66.7 % and 63.8 % at concentrations of 2 and 4 mM.

***Effect of R. × damascena essential oil, MeOH, and different fractions on B16F10 cellular ROS level***

**Figure 5:**
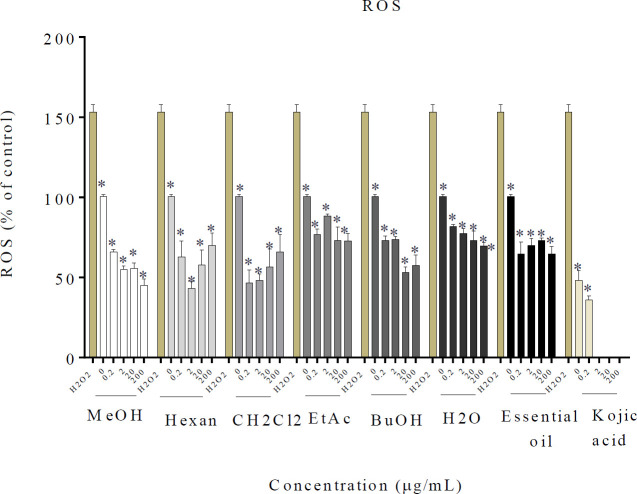
Anti-oxidant effect of *Rosa* × *damascena* essential oil, MeOH, and different fractions on cellular ROS level in B16F10 melanoma cells. The cells were treated with *R. *× *damascena* essential oil, MeOH, and different fractions (0.2–200 µg/ml) or Kojic acid (2.0–4.0 mM). Data are expressed as mean±SD. *P<0.05 compared with control

As can be seen in Figure 5, all essential oils, MeOH, and the different fractions effectively suppressed the H_2_O_2_-induced oxidative stress, while the well containing only H_2_O_2_ increased ROS production.

**Table 1.** *Rosa *× *damascena* essential oil GC-MS analysis. Chemical composition (%) of essential oil of *R.* *damascena *aerial parts

**NO**	**Compound**	**RI ** ^1^	**Percentage %**
1	n-heptanal	901	0.1
2	α-pinene	936	0.6
3	benzaldehyde	962	*t*
4	sabinene	975	0.1
5	β-pinene	977	0.2
6	β-myrcene	993	0.2
7	ortho-cymene	1026	*t*
8	limonene	1030	*t*
9	1,8-cineol	1032	*t*
10	γ-terpinene	1062	*t*
11	linalool	1101	1.1
12	nonanal	1105	0.1
13	cis-rose oxide	1112	0.4
14	phenyl ethyl alcohol	1114	1.4
15	trans-rose oxide	1129	0.2
16	nerol oxide	1158	0.1
17	terpinen-4-ol	1178	0.6
18	α-terpineol	1191	0.4
19	citronellol	1225	37.1
20	unknown	1228	0.2
21	neral	1232	0.5
**22**	geraniol	1255	12.7
23	geranial	1268	0.6
24	methyl geranate	1327	0.1
25	citronelyl acetate	1358	0.8
26	eugenol	1361	1.9
27	neryl acetate	1368	0.1
28	geranyl acetate	1387	1.4
29	β-elemene	1392	0.2
**30**	methyl eugenol	1408	5.0
31	β-caryophyllene	1419	0.6
32	β-copaene	1430	0.1
33	α-guaiene	1440	0.9
**34**	α-humulene	1454	0.6
35	germacrene D	1482	1.5
36	phenylethyl 2- methyl butanoate	1487	0.3
37	pentadecane	1500	0.6
38	trans-β-guaiene	1506	0.5
39	(E,E)-α-farnesene	1510	0.1
40	δ-cadinene	1525	0.1
41	E-nerolidol	1566	0.1
**42**	2-phenyl ethyl tiglate	1587	0.1
**43**	hexadecane	1600	0.1
**44**	γ-eudesmol	1633	0.1
45	*tau*-muurolol	1644	0.1
46	α-eudesmol	1651	0.1
47	α-cadinol	1656	0.3
48	8-heptadecene	1678	0.3
49	*n*-heptadecane	1700	2.3
**50**	Z,E-farnesal	1716	0.1
51	Z,E-farnesol	1724	1.2
52	benzyl benzoate	1765	0.1
53	octadecane	1800	0.3
54	phenyl ethyl benzoate	1854	0.3
55	Z-5-Nonadecene	1876	3.9
**56**	nonadecane	1901	11.8
57	ethyl hexadecanoate	1996	*t*
58	eicosane	2001	1.2
**59**	*n*-heneicosane	2101	5.1
60	*n*-docosane	2200	0.1
**61**	*n*-tricosane	2300	0.9
62	*n*-Tetracosane	2400	0.1
63	*n*-pentacosane	2500	0.2
	**Major Grouped Compounds**		
	Monoterpene hydrocarbons		1.2
	Oxygenated monoterpenes		56.0
	Sesquiterpene hydrocarbone		4.5
	Oxygenated sesquiterpenes		1.8
	Miscellaneous compounds		36.3

1- RI: The Kovats retention indices relative to C8-C20 n-alkanes were determined on CP-Sil 8CB capillary column

t: trace > 0.05%

**Figure 6:**
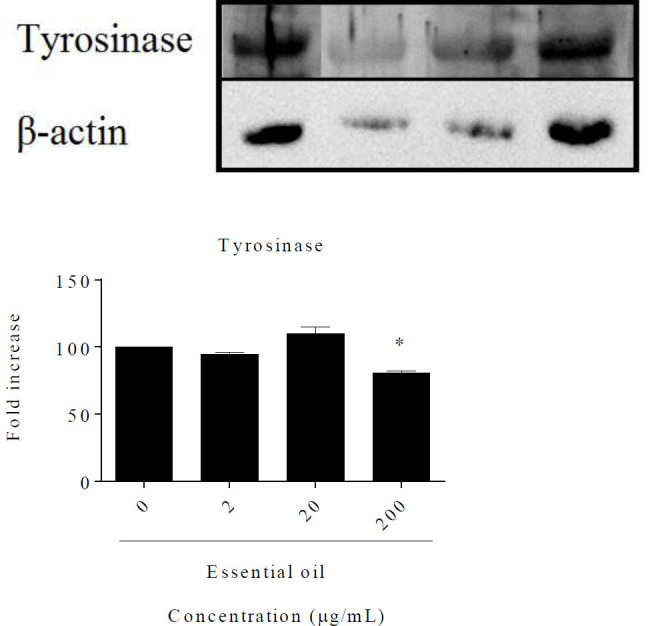
Effect of *Rosa *× *damascena* essential oil on tyrosinase protein level in B16F10 melanoma cells. Cells were treated with *R.* × *damascena* essential oil (2–200 µg/ml). The amount of tyrosinase proteins was normalized using their corresponding β-actin using Quantity One software (Bio-Rad, Hercules, CA, USA). Data are expressed as mean±SD. *P<0.05 compared with control

## Discussion

Based on the available information, the present research is the first study on the effect of essential oil, MeOH, and different fractions of *R. × damascena* on B16F10 murine melanoma cell line and melanin production. We investigated the effect of essential oil, MeOH, and different fractions on cell viability, cellular and mushroom tyrosinase activity, melanin content, and ROS. Western blot was performed on total essential oil to determine the amount of tyrosinase antibody. Following UV exposure, free radicals (FR) and ROS play a major role in the production of lipid (L •) radicals, which induce the destruction of cell membranes and finally cells (26, 27). During skin contact with sunlight, especially its ultraviolet (UV) spectrum, the body’s melanocytes begin to synthesize melanin which has a protective effect on skin cells against mutations, DNA repair process errors, and skin cancers (28). Melanin is a biopolymer that is present in almost all living organisms. In humans, melanin is found mainly in the skin, eyes, and hair, and its distribution causes a variety of color patterns in external tissues. Its photochemical properties allow melanin to play a very important natural protective role in absorbing harmful ultraviolet rays (12). At the beginning of melanin synthesis, tyrosine is oxidized to dopaqinone which is catalyzed by the enzyme tyrosinase. The enzyme tyrosinase is responsible for catalyzing the oxidation of monophenols and diphenols to quinones (29) and its activity depends on the presence of copper (30). However, previous studies have shown that during the process of melanin production and skin care, the enzyme tyrosinase has a major role in the production of melanin and the transfer of produced melanin to adjacent cells (29). Due to the high importance of the tyrosinase enzyme in melanin production, the activity of the enzyme in the presence of essential oils, extracts, and different fractions in B16F10 cells was measured to indirectly decide on the effects of this plant on reducing melanin levels. Tyrosinase inhibitory activity was performed in two separate tests on mushroom tyrosinase also in melanoma cells. In general, the mushroom tyrosinase activity test had an acceptable answer in most concentrations used, but in the cellular tyrosinase activity test, no significant decrease in the amount of this enzyme was observed due to differences in the mechanism of these two tests. Kojic acid is a natural compound derived from the mushroom *Aspergillus, Acetobacter*, and Penicillium and is used in concentrations of 1 to 4% in formulations (31). Kojic acid could significantly reduce the activity of cellular and mushroom tyrosinase enzymes, melanin content, and ROS. Other studies have shown that kojic acid has anti-oxidant properties and scavenging free radicals, reducing the activity of mushroom tyrosinase enzymes and ROS which proves the results of our work (32). Hydroquinone and its derivatives have been associated with skin irritation, contact dermatitis, and skin depigmentation. Another complication of hydroquinone drugs is exogenous ochronosis, which usually occurs in black people after long-term use (33). According to the above, the initial toxicity of the essential oil, MeOH, and different fractions on the survival of B16F10 cells was measured. The essential oil, MeOH, and different fractions did not significantly reduce the percentage of viable cells up to concentrations of 200 μg/ml. Plant anti-oxidants reduce oxidative stress and have protective effects against oxidative stress and related diseases (32). Compounds with anti-oxidant properties reduce the oxidation of tyrosine to dopaqinone (34). Also, they reduce melanin synthesis in the melanogenesis pathway (35). Oxygen free radicals appear to have the main role in cardiovascular disease and the development of cancer. Scientists believe that the gradual accumulation of oxygen-free radicals accelerates the aging process throughout the body, including the skin. Also, the sun induces skin damage mainly due to the effect of oxygen-free radicals. Melanin absorbs free radicals produced by the cell cytoplasm and free radicals produced by UV light in the skin (36). Therefore, substances with anti-oxidant effects reduce the production of ROS and free radicals, also, they can decrease the amount of melanin. The existence of phenolic compounds in the ethanol extract of *R. × damascena*, as well as the high anti-oxidant activity of its extract were proven in the work of Chroho *et al.* (37). Using GC-MS, the oil component of the plant essential oil was determined from *R. × damascene*. As listed in Table 1 the essential oil contains 63 compounds, of which 12.7% geraniol and 37.1% citronellol were the chief components and the majority of compounds in essential oils were 56% oxygen monoterpenes (56%). 

It is shown that geraniol reduces oxidative stress induced by cyclosporine A in rat kidneys. In this way, they decrease Wnt/β-catenin and ROS generation in the cell (38). Methyl-eugenol is identified as a specific anti-oxidant in some important aromatic herbs and spices (39). It is discovered that the great abundance of eugenol and methyl-eugenol in wild *Laurus nobilis* L. leaves is responsible for the higher level of anti-oxidant activities of this plant in comparison with the cultivated one (40). Additionally, it was shown that citronellol, one of the major monoterpenes in *Artemisia scoporis* essential oil, showed strong anti-oxidant and radical scavenging activity (41). In the work of Yasa *et al.*, three flavonols were identified in the ethanol extract of rose with anti-oxidant properties (6). Another study showed that this plant inhibits fat oxidation and can prevent diseases caused by overproduction of free radicals (42). Furthermore, this plant contains vitamin C, which in itself has anti-inflammatory and anti-oxidant properties (43). Wang showed that roses have high amounts of polyphenols, especially flavonols, and possess anti-oxidant and free radical scavenging properties (44). In another study, the essential oil of rose showed an anti-oxidant effect and protected the sex cells exposed to ROS (45). The protective effect of* R. × damascena* on rat ovarian tissue in copper-induced poisoning was demonstrated to be via anti-oxidant effect (46). According to the results, 0.2, 20, and 200 µg/ml of essential oils had a significant effect on reducing melanin levels. As a result, it can be said that *R. × damascena* essential oil decreases the production of the total melanin content of B16F10 cells, mushroom tyrosinase activity, and ROS, which can ultimately reduce melanin production. In one study, the anti-oxidant and protective effects of compounds in citrus essential oils were investigated (47). Another study examined the compounds of 57 species of wild Sicilian rosemary. The predominant compounds identified were investigated and proven (48). GC-MASS analysis of Senecionudicaulis essential oil contains 1.13% citronellol and its anti-oxidant effects have been proven in one study (49). So, considering that the effect of the compounds in *Rosa × damascena* essential oil has been proven in the above studies, it can have a promising therapeutic effect in the treatment of inflammatory skin diseases, skin thickening, and skin brightening.

## Conclusion

We have reported here the antimelanogenic and anti-tyrosinase effect of *R. × damascena *on the B16F10 murine melanoma cell line for the first time. The results indicate *R. × damascena *essential oil may be used as a promising agent in developing new treatments for hyperpigmentation in cosmetics and also in agriculture and food industries as an anti-browning agent.

## Authors’ Contributions

E H and M RK were responsible for investigation, methodology, literature searches, and writing the original draft. SA E, JA, and Z B were responsible for investigation, methodology, literature searches, review, and editing, Z TN was responsible for conceiving, designing, review, and editing.

## Ethics Issues

 This work is carried out on B16F10 murine melanoma cells and there is no need for ethical clearance.

## Conflicts of Interest

 There are no conflicts of interest in this study**.**
